# The Relationship between Obesity, Sleep and Physical Activity in Chinese Preschool Children

**DOI:** 10.3390/ijerph15030527

**Published:** 2018-03-15

**Authors:** Meimei Ji, Amber Tang, Yefu Zhang, Jiaojiao Zou, Guangyu Zhou, Jing Deng, Lina Yang, Mingzhi Li, Jihua Chen, Hong Qin, Qian Lin

**Affiliations:** 1 Department of Nutrition Science and Food Hygiene, Xiangya School of Public Health, Central South University, 110 Xiangya Road, Changsha 410078, China; jimeimei1024@foxmail.com (M.J.); yefuzhang@foxmail.com (Y.Z.); zjj170605@foxmail.com (J.Z.); guany_zhou@163.com (G.Z.); ylnly1997@csu.edu.cn (L.Y.); lmz1976@126.com (M.L.); chenjh@csu.edu.cn (J.C.); qinhong@csu.edu.cn (H.Q.); 2 Department of Molecular, Cellular and Developmental Biology, Yale University, 219 Prospect St, New Haven, CT 06511, USA; amber.t30@gmail.com; 3 Department of Epidemiology and Statistical Science, Xiangya School of Public Health, Central South University, 110 Xiangya Road, Changsha 410078, China; jingdeng@csu.edu.cn

**Keywords:** preschool children, overweight, obesity, sleep, physical activity

## Abstract

Background: Pediatric overweight and obesity has become a major public health problem in China. The goal of this study is to understand overweight and obesity in preschool children in Changsha City in the context of their sleep and physical activity. These results offer feasible proposals to reduce levels of overweight and obesity among preschool children. Methods: A total of 112 preschoolers aged three to six years old were investigated using multiple stage stratified cluster sampling and simple random sampling. Questionnaires were used to collect general information about children and their families. Body mass index (BMI) was used as an indicator of overweight and obesity. Age- and sex-specific cutoff values for Chinese children and adolescents were used to determine child weight status. Children’s sedentary time was reported by caregivers, while physical activity and sleep were recorded using fitness bracelets (Misfit Shine 2). Results: The prevalence of childhood overweight and obesity were 15.2% and 9.8% respectively. Preschool-aged children travelled 11,111 ± 3357 and 10,350 ± 2973 steps per day on weekdays and weekends respectively. The number of daily steps was not statistically different between weekdays and weekends. The amount of time spent daily doing vigorous activity on weekdays and weekends was significantly different, with an average time of 20.5 ± 31.6 min and 10.3 ± 15.3 min respectively (*p* = 0.002). Furthermore, 10.7% and 50.9% of children used screens for more than two hours on weekdays and weekends respectively (*p* < 0.001). Children slept for significantly longer on weekends (8.3 ± 0.9 h) than on weekdays (8.1 ± 0.7 h) (*p* = 0.037). A significantly higher proportion of students also fell asleep before 10:00 p.m. on weekends (26.8%) compared to weekdays (15.2%) (*p* < 0.001). Parent’s BMI values were positively correlated with child BMI, the monthly household income was negatively associated with child BMI. Male children were more likely to have a higher BMI than female children. Children who were obese were also more likely to have shorter sleep times compared to children of normal weight (*p* = 0.047). Conclusions: There was a high prevalence of overweight and obesity among the Chinese preschool children in this study. Students also demonstrated poor sleep and physical activity habits. Future research is necessary to explore the relationship between sleep, physical activity and weight status for young children in China.

## 1. Introduction

Over the past two decades, the global prevalence of overweight and obesity in preschool children has risen sharply. According to a World Health Organization (WHO) survey, about 41 million children under the age of five were affected by overweight or obesity in 2016. Furthermore, half of overweight or obese children lived in Asia [1]. In 2013, the prevalence of overweight and obesity for children under six years old in China was 8.4% and 3.1% respectively, and this prevalence has continued to increase [2]. Furthermore, maintaining a healthy weight in childhood is particularly important given that childhood overweight and obesity can be detrimental towards physical, psychological, and social development. Overweight and obesity in childhood is also closely related to cardiovascular and cerebrovascular diseases in adulthood, including diabetes, metabolic syndrome, and cancer [3]. Therefore, understanding factors associated with childhood overweight and obesity is critical for addressing this pressing public health concern. 

Due to China’s rapid economic development, people’s standards of living have vastly improved and their lifestyles have undergone tremendous changes over the past few decades. However, China’s rising levels of overweight and obesity have also been attributed to the country’s recent socioeconomic growth [4]. Levels of physical activity have decreased as people rely less on biking and walking for transportation. Sleep quantity and quality have also been affected by increased use of electronic devices before sleep [5]. 

Current studies on the effects of sleep on overweight and obesity are mainly focused on sleep duration. Sleeping less than nine hours a day has been reported to be a risk factor for childhood overweight and obesity in China [6,7,8]. In addition, a Chinese study showed school-age children who slept less did more light physical activity, and children with earlier sleep time showed less sedentary activity [9]. Insufficient physical activity has also been linked to overweight and obesity among children [10]. Furthermore, out of various demographic factors, only socioeconomic status was correlated with physical activity [11]. Studies in China have suggested that the family environment has a large impact on physical activity among preschool children. Furthermore, parental athletic skill and perception of child weight are associated with child physical activity [12].

Developing good habits in early childhood is crucial to health throughout childhood, adolescence and adulthood [13,14]. However, little research has been conducted on sleep and physical activity among young children aged three to six years old in China in the context of overweight and obesity. Preschool children are of particular concern as they are in a critical period of growth and development. Therefore, understanding the association between physical activity, sleep and body mass index (BMI) in this group is important for informing future strategies to prevent obesity. Here, we aimed to characterize and describe sleep and physical activity among three- to six-year-old preschool children in Changsha, China. The goal of this study was to better understand the impact of sleep and physical activity on weight status in Chinese children and to suggest effective methods for reducing levels of childhood overweight and obesity.

## 2. Methods

### 2.1. Sampling

The investigation was carried out from July to October 2017 in Changsha, which is the capital of the Hunan Province in China. A multiple stage stratified cluster sampling and a simple random sampling were used to recruit participants for the study. First, two districts were selected from 6 districts of Changsha. Then, six preschools were randomly selected from 298 public preschools with at least 300 children in these two districts. All six of the randomly selected preschools agreed to participate in this study. Each preschool consisted of three grades—the lower, middle, and upper level classes. We randomly selected one class from each grade and ten children in each class.

### 2.2. Ethical Approval

Ethical approval was granted by the Xiangya School of Public Health IRB, in Central South University (No. XYGW-2017-21). Before the investigation, written informed consent by caregivers was obtained and all information was kept strictly confidential.

### 2.3. Data Collection

The survey included a self-administered caregiver questionnaire, physical measurements of the children, and data collected from fitness wristbands. The questionnaire included 46 items, which were adapted from a previous study [15], consisted of four parts: (1) General information about the child, his/her parents and caregivers: sex, birthdate, birth weight, whether preterm or not, height and weight of the child and his/her parents, parenthood and who is the primary caregiver, etc.; (2) Socio-economic status (SES): family income, family size, parents’ education levels and career, etc.; (3) Sedentary time: questions about children’s sedentary time throughout the week were investigated. Sedentary time included screen time (use of mobile phones, tablets, etc.) and other sitting time (reading, doing homework, drawing, etc.). Height and weight measurements from annual physical examinations conducted by each school were used to calculate student BMI. The age of each student was calculated from the date at which physical measurements were collected. Age- and sex-specific BMI cutoffs developed for Chinese populations were used to classify participant weight status [16]. 

We sent the questionnaire to the preschool on Thursday and told the head teachers how to fill out the questionnaire. The head teachers gave the questionnaires to the valid caregivers when they picked up the children on Thursday or Friday afternoon, with oral instruction on how to fill out the questionnaire and the date of collection. The valid caregivers refer to a person who primarily cares for the children in a family. If caregivers had problems when they filled out the questionnaire, they were asked to return it to the head teacher. If the parents completed the questionnaire, we provided them with feedback on children’s sleep and physical activity as compensation, by comparing their responses with the recommended values, we gave each child’s sleep and physical activity evaluation and specific recommendations.

Misfit Shine 2 (Misfit Inc., Apple Inc., Burlingame, CA, USA) fitness wristbands were used in our study to monitor the sleep and physical activity of each student. Bracelets were worn Thursday through Monday to record four nights of sleep and three days of physical activity. Hours of sleep and number of steps per day were compared to recommended values for 3 to 5-year-old children from The National Sleep Foundation and the Canadian Society for Exercise Physiology (8–13 h and 8000 steps per day respectively) [17]. 

### 2.4. Statistical Analysis

EpiData 3.1 software (The EpiData Association, Odense, Denmark) was used for data entry and the IBM SPSS 18.0 software package (IBM Corp., Armonk, NY, USA) was used for data analysis. Descriptive information was presented as a percentage or as mean ± standard deviation. Chi-squared tests and one-way ANOVA were used to analyze general demographic data for different BMI categories. Chi-squared tests and *t*-tests were also used to examine differences in physical activity and sleep between weekdays and weekends. Multiple linear regression was used to identify factors that might influence child BMI. Binary logistic regression analysis was used to analyze the relationship between sleep, BMI and physical activity. All statistical tests were two-sided and *p*-values < 0.05 were considered statistically significant.

## 3. Results

### 3.1. General Characteristics of Study Population

In total, 112 preschool students were included in the final analysis. There was a similar distribution of males and females among the participants (55 male, 57 female). Most of the children surveyed were Han Chinese (93.8%), aged four to five years old (44.6%), breastfed (88.4%) and the only child (67.9%). More than 60% of children had four to five members in their households. About 80% of households had a monthly income of more than 9000 RMB. Fathers or mothers were the most common primary caregivers (68.8%), followed by grandparents (30.4%). Over half of parents had above a college level education (68.8% of mothers and 78.6% of fathers). The prevalence of childhood overweight and obesity were 15.2% and 9.8%, respectively (Table 1). The rate of overweight and obesity among children who were primarily cared for by their parents was significantly higher than that of children cared for by grandparents (*p* = 0.043). 

### 3.2. Comparison of Physical Activity and Sleep on Weekdays and Weekends

Table 2 shows that preschool-aged children significantly spent more time doing vigorous activity on weekdays (20.5 ± 31.6 min) than on weekends (10.3 ± 15.3 min) (*p* = 0.002). However, there was no significant difference detected for other physical activity indicators. Children’s daily screen time, other sedentary time and hours spent sleeping were also significantly lower during the weekdays than the weekends (*p* < 0.001, *p* < 0.001, *p* = 0.037 respectively). The proportion of preschoolers who fell asleep before 10:00 p.m. on weekdays (15.2%) was significantly lower than those who slept before 10:00 p.m. on the weekends (26.8%) (*p* < 0.001).

### 3.3. Factors Associated with BMI in Preschool Children

Results from a multiple linear regression analysis of factors associated with children’s BMI are presented in Table 3. The results showed that parent’s BMI values were positively correlated with child BMI, the monthly household income was negatively associated with child BMI. Male children were more likely to have a higher BMI than female children.

### 3.4. The Relationship between Sleep, BMI and Physical Activity

Sleep time was divided into two categories (<8 h and 8–13 h. No subjects in this study sleep time reached 13 h, so we did not consider this situation) according to the National Sleep Foundation recommendations for three- to five-year-old children. The results of binary logistic regression analysis in Table 4 showed that childhood obesity was negatively associated with their sleep times, that is, obese children were more likely to sleep less than eight hours (*p* = 0.047).

## 4. Discussion

This study found that the prevalence of overweight and obesity were 15.2% and 9.8% respectively among preschool children in Changsha. The prevalence of obesity among preschool children was a little higher than the 9.4% prevalence reported in the United States between 2013 and 2014 [1]. The overweight levels found here were also higher than the prevalence previously reported in nine Chinese districts in 2012, where 12.2% of survey participants were overweight [18]. The overweight values reported here were also higher than the prevalence in a 2008 study in Changsha (3.9%) [19]. However, a study conducted in six cities in northern China found a similar prevalence of overweight and obesity in children two to seven years old [20]. The high levels of overweight and obesity among preschool children in Changsha are concerning and their development trend is not optimistic. 

The results showed that parent’s BMI values were positively correlated with child BMI, the monthly household income was negatively associated with child BMI and male children were more likely to have a higher BMI than female children, which was consistent with other findings [21,22]. This may be attributable to the different body standards for boys and girls as well as differences in physical requirements by gender. For example, caregivers may think that boys should eat more to grow faster, which encourages overeating and can lead to obesity. High monthly household income could indicate that parents have a relatively high level of education which might be associated with them having a better understanding of children’s weight and, together, these factors could help to prevent overweight and obesity in children. We also found that parental obesity was closely related to child obesity. This relationship can be explained through both genetic and lifestyle factors passed on from parents to their children. This suggests that early interventions for childhood obesity should focus on children of parents with overweight or obesity.

At present, most research on the recommended amount of physical activity in China is aimed at adolescents or adults. We used recommendations from the Canadian Society for Exercise Physiology for children three to five years of age. These guidelines suggested 8000 steps per day for preschool children with at least 180 min of total physical activity and 60 min of moderate to vigorous activity daily [17]. A number of foreign countries have developed manuals for preschool children’s physical activity, but the recommendations for physical activity time and intensity have not been unified [23,24]. In this study, children did not spend enough time doing physical activity and more than half spent more than two hours of screen time during the weekend. Long screen time not only increases the risk of overweight and obesity, but also is associated with poor vision, cervical vertebra development and cognitive development [25]. 

On average, we found that preschool-age children spent significantly more time doing vigorous activity on weekdays than weekends. Greater overall physical activity on the weekdays may be due to Chinese governmental management of preschools. As of March 2016, preschool regulations required that children’s outdoor activity time (including outdoor sports time) be at least two hours per day. In the absence of this requirement on weekends, children may have greater screen usage and less physical activity. Training and educational initiatives may improve caregiver awareness and monitoring of their children’s physical activity.

The average sleep time per night of children in this survey was 8.2 ± 0.7 h, which was lower than the 9.9 ± 0.7 h reported in children aged three to six years old in Wuhu City [26]. In most previous studies on children’s sleep time, data was self-reported by parents [10,26]. As a result, parents would likely estimate sleep time from the point at which children went to bed, which was likely an overestimate of the time actually asleep. In the course of our investigation, some parents reflected that their children did not immediately go to sleep after they went to bed.

Overall, children went to bed earlier and slept longer on weekends, but the duration of deep sleep did not increase. In China, preschools typically have a regular two hour lunch break and nap for children. On weekends, however, children’s lunch breaks and nap times are irregular. Children may not nap in the afternoons causing them to be tired earlier in the evening, leading to an early sleep time. However, reduced physical activity and increased sedentary time on weekends may account for the lack of difference in deep sleep throughout the week. Currently, there is only one recommended value for sleep among school-aged children in China, and no existing recommendation for preschool children. The National Sleep Foundation recommends 10–13 h of sleep per night for three- to five-year-old children; however anywhere from 8–14 h of sleep may be appropriate. However, 34.8% of the children included in our study failed to meet the recommended amount of sleep for their age group. To improve consistency of children’s daily sleep schedules, preschools and caregivers should strengthen communication when arranging children’s sleep times. 

We also found that children who were obese were more likely to have shorter sleep times. However, the current causal relationship between childhood obesity and sleep remains unclear. On the one hand, obesity is a risk factor for obstructive sleep apnea/hypopnea syndrome (OSAHS), which can cause frequent wakefulness, insufficient sleep, fatigue during the day, decreased exercise and increased food intake [27]. On the other hand, past studies have shown that lack of sleep and sleeping late among children are risk factors for overweight or obesity [21,28,29]. The mechanism of this relationship is not clear, however, existing research has suggested that it may be related to hormones and physical activity [30]. Changes in the levels of hormones, such as leptin, ghrelin, insulin, and cortisol, can activate sympathetic nervous activity in the brain, increasing dietary intake and resulting in excess energy. Too little sleep may also cause children to sleep more during the day, thus reducing their daytime activity and causing weight gain. Since insufficient sleep is a modifiable risk factor, these findings are instructive in the clinical prevention and treatment of childhood obesity.

In addition, it has been suggested that physical activity is positively correlated with sleep, and screen time is negatively correlated with sleep [10]. However, we have yet to find a relationship between children’s daily physical activity and sleep time. This may be due to the small sample size of our study, or measurement error associated with the fitness wristband. The Misfit Shine could not accurately track activity when participants walked straight indoors, which may have led to underestimation of the actual number of steps [31]. 

In view of the current status of overweight and obesity, sleep and physical activity among preschool children in Changsha, China, we suggest the following measures. First, strengthen health education efforts to improve health awareness about overweight and obesity among caregivers and preschools. Preschools and families should regularly monitor children’s height and weight, detect abnormal weight and implement early interventions. Secondly, encourage schools to educate caregivers on healthy behaviors for young children to encourage the development of good sleep and physical activity habits. Lastly, further work is necessary to establish healthy standards for sleep and physical activity suitable for preschool children in China. These standards would provide guidance for families with young children when establishing healthy sleep and physical activity behaviors.

This study used a written questionnaire combined with fitness tracking wristbands to understand preschool children’s sleep and physical activity. It is one of few studies currently available that use fitness tracking devices to investigate physical activity and sleep. Previous studies have used pedometers or accelerometers to investigate physical activity. However, these tools require daily adult assistance to disassemble and assemble them, which may be troublesome. A comparison of the accuracy and stability of wearable fitness devices showed that Misfit outperformed three other devices on the stairs and treadmill, with an accuracy of 97.8% and repeatability of 0.8 [31]. In addition, the Misfit Shine 2 sports bracelet only needed to be worn on the wrist of the child at the beginning of the investigation and removed at the end. There was no need to take it off at any time during the investigation. 

There were several limitations in our study. First, our survey was a cross-sectional study that did not define the causal relationship between obesity and sleep; Second, the sample size was modest and other factors might have impacted the accuracy and authenticity of the results, such as diet history and weight changes, so the findings should be viewed with caution; Lastly, the bracelet itself may have had measurement errors. Additional studies with large samples or cohort studies are needed to explore the causal relationship and mechanisms of overweight and obesity, sleep and physical activity.

## 5. Conclusions

Chinese preschool children had a high prevalence of overweight and obesity, and demonstrated poor sleep and physical activity habits. Additional research is necessary to further explore the mechanism of influence among overweight and obesity, sleep and physical activity for young children in China.

## Figures and Tables

**Table 1:** Socio-demographic characteristics of study population (*n*, %).

Variables	Normal Weight (*n* = 84)	Overweight (*n* = 17)	Obesity (*n* = 11)	Total (*n* = 112)	F/Chi-sq Value	*p*
Sex					2.874	0.239
Male	40 (72.7%)	7 (12.7%)	8 (14.5%)	55 (49.1%)		
Female	44 (77.2%)	10 (17.5%)	3 (5.3%)	57 (50.9%)		
Ethnicity					0.728	0.806
Han	79 (75.2%)	16 (15.2%)	10 (9.5%)	105 (93.8%)		
Minorities	5 (71.4%)	1 (14.3%)	1 (14.3%)	7 (6.3%)		
Age					2.287	0.711
3–4 years	28 (82.4%)	3 (8.8%)	3 (8.8%)	34 (30.4%)		
4–5 years	37 (74%)	8 (16.0%)	5 (10.0%)	50 (44.6%)		
5–6 years	19 (67.9%)	6 (21.4%)	3 (10.7%)	28 (25.0%)		
Breastfed					3.250	0.144
Yes	75 (75.8%)	13 (13.1%)	11 (11.1%)	99 (88.4%)		
No	9 (69.2%)	4 (30.8%)	0 (0%)	13 (11.6%)		
The only child					10.581	0.005
Yes	60 (78.9%)	6 (7.9%)	10 (13.2%)	76 (67.9%)		
No	24 (66.7%)	11 (30.6%)	1 (2.8%)	36 (32.1%)		
Family Size					10.155	0.027
≤3	31 (73.8%)	3 (7.1%)	8 (19.0%)	42 (37.5%)		
4–5	52 (76.5%)	13 (19.1%)	3 (4.4%)	68 (60.7%)		
≥6	1 (50.0%)	1 (50%)	0 (0%)	2 (1.8%)		
Monthly household income Quartile					10.442	0.068
<7000 RMB (Bottom)	6 (54.5%)	2 (18.2%)	3 (27.3%)	11 (9.8%)		
7000–9000 RMB (3rd)	7 (63.6%)	1 (9.1%)	3 (27.3%)	11 (9.8%)		
9000–11,000 RMB (2nd)	23 (71.9%)	7 (21.9%)	2 (6.3%)	32 (28.6%)		
≥11,000 RMB (Top)	48 (82.8%)	7 (12.1%)	3 (5.2%)	58 (51.8%)		
Primary Caregiver relationship to children					9.012	0.043
Father or Mother	57 (74.0%)	9 (11.7%)	11 (14.3%)	77 (68.8%)		
Grandparents	26 (76.5%)	8 (23.5%)	0 (0%)	34 (30.4%)		
Nanny or others	1 (100%)	0 (0%)	0 (0%)	1 (0.9%)		
Mother’s BMI *	20.8 ± 2.4	21.7 ± 2.7	22.4 ± 3.6	21.1 ± 2.6	2.514	0.086
Father’s BMI *	23.8 ± 2.5	25.0 ± 2.6	24.4 ± 2.7	24.0 ± 2.5	1.800	0.170
Mother‘s education level					3.731	0.747
Junior middle school	1 (100.0%)	0 (0%)	0 (0%)	1 (0.9%)		
High school	5 (83.3%)	1 (16.7%)	0 (0%)	6 (5.4%)		
College	23 (82.1%)	2 (7.1%)	3 (10.7%)	28 (25.0%)		
>College	55 (71.4%)	14 (18.2%)	8 (10.4%)	77 (68.8%)		
Father‘s education level					1.993	0.712
High school	6 (85.7%)	1 (14.3%)	0 (0%)	7 (6.3%)		
College	15 (17.9%)	1 (5.9%)	1 (9.1%)	17 (15.2%)		
>College	63 (71.6%)	15 (17.0%)	10 (11.4%)	88 (78.6%)		
Total	84 (75.0%)	17 (15.2%)	11 (9.8%)	112 (100%)		

* The parents’ BMI analysis used one-way ANOVA, and the others used Chi-square tests.

**Table 2:** Daily physical activity, screen time and sleep during the weekdays and weekends (*n* = 112).

Variables	Weekday	Weekend	Chi-sq/*t*-Test Value	*p*
Physical Activity				
Daily steps	11,111 ± 3357	10,350 ± 2973	1.795	0.074
Daily steps ≥8000 ^®,^^a^ (*n*, %)	93 (83.0%)	91 (81.3%)	0.122	0.727
Daily light activity (minutes)	26.8 ± 22.3	21.9 ± 18.5	1.805	0.072
Daily moderate activity (minutes)	27.2 ± 30.8	29.3 ± 29.4	−0.512	0.609
Daily vigorous activity (minutes)	20.5 ± 31.6	10.3 ± 15.3	3.092	0.002
Daily MVPA time >60 min *^,a^ (*n*, %)	33 (29.5%)	21 (18.8%)	3.514	0.061
Sedentary time				
Daily screen time (minutes)	44.8 ± 42.1	124.1 ± 98.3	−7.847	<0.001
Daily other sedentary time (minutes)	33.5 ± 22.5	65.8 ± 49.6	−6.259	<0.001
Daily screen time >2 h ^#^^,a^ (*n*, %)	12 (10.7%)	57 (50.9%)	42.412	<0.001
Sleep				
Daily sleep duration (hours)	8.1 ± 0.7	8.3 ± 0.9	−2.098	0.037
Daily light sleep (hours)	4.3 ± 0.8	4.5 ± 0.9	−1.936	0.054
Daily deep sleep (hours)	3.8 ± 0.8	3.9 ± 0.8	−0.604	0.546
Sleep duration 8–13 h ^φ,^^a^	64 (57.1%)	69 (61.6%)	0.463	0.496
Bed time				
Fell asleep before 10:00 p.m. ^a^	17 (15.2%)	30 (26.8%)	76.477	<0.001

^a^ Differences were determined by Chi-square tests, and the others used *t*-tests; ^®^ Recommended by the Canadian Society for Exercise Physiology; * Recommended by National Association for Sport and Physical Education (NASPE) in 2011. ^#^ American Academy of Pediatrics (AAP) recommended less than three hours per day of daily screen time for preschool children in 2001; ^φ^ The National Sleep Foundation suggests that eight to nine hours of sleep may be appropriate for toddlers, although 10–13 h is recommended (only one case had a sleep duration above 10 h, so we combined these recommendations).

**Table 3:** Factors associated with BMI among preschool children identified by a multiple linear regression (*n* = 112).

Variable	Coefficient (Crude)	Std. Err.	Coefficient (Adjusted)	*t*	*p*
Sex (male = 1, female = 0)	0.750	0.296	0.248	2.535	0.013
Monthly household income	−2.245	0.123	−0.182	1.991	0.049
Father’s BMI	0.117	0.058	0.194	2.000	0.048
Mother’s BMI	0.141	0.059	0.243	2.380	0.019
Constant	10.672	2.225		4.796	0.000

**Table 4:** Binary logistic regression coefficients (95% CI) for sleeping an appropriate or recommended length of time (8–13 h) (*n* = 112).

Variables	Daily Sleep Duration Maybe Appropriate 8–9 h or Recommended 10–13 h					
	B	S.E.	Wald χ^2^	Df	*p*	OR (95% CI)
Daily MVPA (minutes)	−0.307	0.692	0.197	1	0.657	0.735 (0.189–2.855)
Daily screen times (minutes)	−0.967	0.646	2.242	1	0.134	0.380 (0.107–1.348)
Children’s BMI						0.144 (0.034–0.601)
Normal			3.966	2	0.138	
Overweight	−0.214	0.605	0.125	1	0.724	0.808 (0.247–2.642)
Obesity	−1.605	0.807	3.955	1	0.047	0.201 (0.041–0.977)
Constant	2.145	0.998	4.622	1	0.032	8.542

Adjusted for age, gender, father’s BMI, mother’s BMI, total physical activity time, daily steps and daily sedentary time. B: un-standardized beta coefficient; S.E.: Standard error; Df: Degree freedom; OR: Odds Ratio; CI: Confidence interval.

## References

[1] WHO Releases Guidelines to Address Overweight and Obesity in Children. http://www.who.int/nutrition/topics/new-release-guideline-obesity-children/en/.

[2] Gu J.F. (2016). Interpretation of Nutrition and Chronic Diseases in Chinese People (2015). Acta Nutr. Sin..

[3] Zhang Y.R., Yang L.Q. (2015). Research Advances in Childhood Obesity. Med. Recapitulate.

[4] Xue H.M., Liu Y., Duan R.N. (2014). Prevalence and Related Factors of Overweight and Obesity in Chinese Children and Adolescents. Chin. J. Sch. Health.

[5] Caitlyn F., Eric L., Steven H. (2017). Bedtime Use of Technology and Associated Sleep Problems in Children. Glob. Pediatr. Health.

[6] Meng L.P., Liu A.L., Hu X. (2012). Report on Childhood Obesity in China (10): Association of Sleep Duration with Obesity. Biomed. Environ. Sci..

[7] Shan X.Y., Xi B., Cheng H., Hou D.Q., Wang Y., Mi J. (2010). Prevalence and Behavioral Risk Factors of Overweight and Obesity among Children Aged 2–18 in Beijing, China. Int. J. Pediatr. Obes..

[8] Jiang F., Li S.H., Shen D.M., Li S.H., Shen X.M. (2010). Study on the Correlation between Sleep Deprivation and Obesity in Children. Chin. J. Child Health Care.

[9] Song Y.J. (2015). Serial Studies on the Effect of Sleep on Eating Habits and Physical Activity in Children.

[10] Anna S., Marjory K., Pieter J.S. (2015). Television, Sleep, Outdoor Play and BMI in Young Children: The GECKO Drenthe Cohort. Eur. J. Pediatr..

[11] Vorwerg Y., Petroff D., Kiess W., Blüher S. (2013). Physical Activity in 3–6-Year Old Children Measured by SenseWear Pro^®^: Direct Accelerometry in the Course of the Week and Relation to Weight Status, Media Consumption, and Socioeconomic Factors. PLoS ONE.

[12] Russell H.P., Jennifer R.O., William H.B., Kerry L.M., Erin K.H., Marsha D. (2015). Top 10 Research Questions Related to Physical Activity in Preschool Children. J. Beijing Sport Univ..

[13] Matusik P., Malecka-Tendera E. (2011). Overweight Prevention Strategies in Preschool Children. Int. J. Pediatr. Obes..

[14] Skouteris H., Mccabe M., Swinburn B., Hill B. (2010). Healthy Eating and Obesity Prevention for Preschoolers: A Randomised Controlled Trial. BMC Public Health.

[15] Lin Q., Peymané A., Karla H. (2015). Health Allowance for Improving the Nutritional Status and Development of 3–5-Year-old Left-behind Children in Poor Rural Areas of China: Study Protocol for a Cluster Randomised Trial. Trials.

[16] Li H., Zong X.L., Ji C.Y., Mi J. (2010). Body Mass Index Cut-offs for Overweight and Obesity in Chinese Children and Adolescents aged 2–18 Years. Chin. J. Epidemiol..

[17] Zhang J.Y. (2016). Daily Step Count Target for Meeting Activity Guidelines in Shanghai Preschoolers.

[18] Wang F.M., Jin X., Jiang J.X., Yao Q., Yang Q. (2017). Situation and Effecting Factors of Preschool Children among Several Cities in China. Chin. J. Child Health Care.

[19] Kang R.T., Zhong Y., Luo H.Y., Jiang Y.H., Liu K.X., You C. (2008). Epidemiologic Status and Influencing Factors of Simple Obesity in Preschool Children. Chin. J. Child Health Care.

[20] Ma Y.N., Chen T., Wang D., Liu M.M., He Q.C., Dong G.H. (2011). Prevalence of Overweight and Obesity among Preschool Children from Six Cities of Northeast China. Arch. Med. Res..

[21] Hou R.X. (2016). Overweight and Obesity in Preschool Children in Qinhuai District, Nanjing. Jiangsu J. Prev. Med..

[22] Guo J.H., Yan X.J., Qu J.X., Liu S.W. (2017). Evalution on Height and Weight among Left-behind Children Aged ≤5 Years in Rural Areas in Hebei Province. Chin. J. Public Health.

[23] Goldfield G.S., Harvey A., Grattan K., Adamo K.B. (2012). Physical Activity Promotion in the Preschool Years: A Critical Period to Intervene. Int. J. Environ. Res. Public Health.

[24] Vale S., Trost S.G., Duncan M.J., Mota J. (2015). Step Based Physical Activity Guidelines for Preschool-aged Children. Prev. Med..

[25] Katherine L.D., Trina H., Jo S., Jill A.H., Kylie D.H. (2017). Do the Correlates of Screen Time and Sedentary Time Differ in Preschool Children?. BMC Public Health.

[26] Yu M., He H.Y., Zhu L.J. (2017). A Study on the Relationship between Sleep and Obesity in Children Aged 3–6 Years in Wuhu City. Chin. Prim. Health Care.

[27] Yin S., Chen Z.R. (2013). Correlation of Simple Obesity Body Mass Index and Obstructive Sleep Apnea Hypopnea Syndrome in Children. Chin. Community Doc..

[28] Landhuis C.E., Poulton R., Welch D., Hancox R.J. (2008). Childhood Sleep Time and Long-term Risk for Obesity: A 32-Year Prospective Birth Cohort Study. Pediatrics.

[29] Chaput J.P., Brunet M., Tremblay A. (2006). Relationship between Short Sleeping Hours and Childhood Overweight/Obesity: Results from the ‘Québec en Forme’ Project. Int. J. Obes..

[30] Chaput J.P., Després J.P., Bouchard C., Tremblay A. (2007). Short Sleep Duration is Associated with Reduced Leptin Levels and Increased Adiposity: Results from the Quebec Family Study. Obesity.

[31] Kanitthika K., Soochan K. (2016). A Comparison of Wearable Fitness Devices. BMC Public Health.

